# Postal recruitment for genetic studies of preterm birth: A feasibility study

**DOI:** 10.12688/wellcomeopenres.15207.2

**Published:** 2020-06-30

**Authors:** Oonagh E. Keag, Lee Murphy, Aoibheann Bradley, Naomi Deakin, Sonia Whyte, Jane E. Norman, Sarah J. Stock

**Affiliations:** 1 Simpson Centre for Reproductive Health, Royal Infirmary of Edinburgh, Edinburgh, EH16 4SA, UK; 2 Edinburgh Clinical Research Facility, Western General Hospital, Edinburgh, EH4 2XU, UK; 3 Queen Margaret Hospital, Dunfermline, KY12 0SU, UK; 4 Department of Clinical Neurosciences, University of Cambridge, Cambridge, CB2 0QQ, UK; 5 MRC Centre for Reproductive Health, University of Edinburgh, Edinburgh, EH16 4TJ, UK; 6 Faculty of Health Sciences, University of Bristol, Bristol, BS8 1UD, UK; 7 Usher Institute of Population Health Sciences and Informatics, University of Edinburgh, Edinburgh, EH16 4UX, UK

**Keywords:** premature birth, fetal membranes, premature rupture, obstetric labour, premature, surveys and questionnaires, phenotype, DNA

## Abstract

**Background: **Preterm birth (PTB) represents the leading cause of neonatal death. Large-scale genetic studies are necessary to determine genetic influences on PTB risk, but prospective cohort studies are expensive and time-consuming. We investigated the feasibility of retrospective recruitment of post-partum women for efficient collection of genetic samples, with self-collected saliva for DNA extraction from themselves and their babies, alongside self-recollection of pregnancy and birth details to phenotype PTB.

**Methods: **708 women who had participated in the OPPTIMUM trial (a randomised trial of progesterone pessaries to prevent PTB [ISRCTN14568373]) and consented to further contact were invited to provide self-collected saliva from themselves and their babies. DNA was extracted from Oragene OG-500 (adults) and OG-575 (babies) saliva kits and the yield measured by Qubit. Samples were analysed using a panel of Taqman single nucleotide polymorphism (SNP) assays. A questionnaire designed to meet the minimum data set required for phenotyping PTB was included. Questionnaire responses were transcribed and analysed for concordance with prospective trial data using Cohen’s kappa ( *k*).

**Results: **Recruitment rate was 162/708 (23%) for self-collected saliva samples and 157/708 (22%) for questionnaire responses. 161 samples from the mother provided DNA with median yield 59.0µg (0.4-148.9µg). 156 samples were successfully genotyped (96.9%). 136 baby samples had a median yield 11.5µg (0.1-102.7µg); two samples failed DNA extraction. 131 baby samples (96.3%) were successfully genotyped. Concordance between self-recalled birth details and prospective birth details was excellent ( *k*>0.75) in 4 out of 10 key fields for phenotyping PTB (mode of delivery, labour onset, ethnicity and maternal age at birth).

**Conclusion: **This feasibility study demonstrates that self-collected DNA samples from mothers and babies were sufficient for genetic analysis but yields were variable. Self-recollection of pregnancy and birth details was inadequate for accurately phenotyping PTB, highlighting the need for alternative strategies for investigating genetic links with PTB.

## Introduction

Preterm birth is the leading cause of neonatal morbidity and mortality, resulting in an estimated economic burden to the public sector in England and Wales in excess of £2.9 billion over 18 years ^1^. Spontaneous preterm birth (PTB) refers to birth less than 37 weeks gestation after the spontaneous onset of contractions ^2^. In England in 2011/2012 27,509 babies were spontaneous PTBs, of which 11,480 were less than 32 weeks gestation ^3^. Although our knowledge surrounding PTB and thus our ability to treat and prevent it has been increasing over time, 95% of preterm births are intractable to current therapies ^4^. Thus, further research into the pathogenesis of PTB is required to decrease this public health problem.

Research has shown that genetic factors contribute to spontaneous PTB. The strongest risk factor for PTB is a history of PTB ^2, 5^, with a recurrence rate after one spontaneous PTB of 15%, which further increases the earlier the gestation, suggesting a maternal genetic component to the risk. Another suggestion of genetic association is the significant ethnic differences in incidence of PTB, with higher rates in women classified as black, African-American and Afro-Caribbean compared with white women, even when environmental confounding factors are taken into consideration ^2^. A familial predisposition has also been shown, with women who were born preterm being more likely to have preterm babies themselves ^6^, as well as women being more likely to have PTB if their sisters have had PTB ^7, 8^.

Recent advances in genetic and bioinformatic technologies now provide the potential to understand the complex interaction of genetic and environmental factors. However, studies of genetic associations with pregnancy complications are dependent on very large numbers of good quality DNA samples from well-phenotyped cases, preferably with samples from mother and baby pairs. Studies of genetic associations with conditions such as breast cancer and diabetes have successfully used postal recruitment, with participants donating DNA through provision of saliva samples, which can be returned by post ^9^. This method could be an efficient way of sample collection from women who have had a PTB and their babies. However, it is crucial to know whether high quality phenotypic information can be provided alongside this approach. An international collaboration of researchers interested in genetic epidemiology studies of pregnancy has highlighted prerequisite phenotypic information essential for performing genetic association studies of preterm birth ^10^. The group highlights the important differences between spontaneous PTB (spontaneous onset of contractions), spontaneous PTB with preterm premature rupture of membranes (PPROM), and medically indicated PTB (medical indications being fetal compromise, such as small for gestational age, or maternal compromise, for example severe pre-eclampsia) ^10^. The genetic associations and pathophysiology underlying these three conditions vary greatly ^10^, hence the necessity for accurate phenotyping in genetic studies in this field specifically. Furthermore, it has not yet been established if maternal recall would be of sufficient quality to support such research. It is also unknown whether postal recruitment and self-collection of samples from mothers, and maternal collection of samples from infants, would be acceptable to participants or yield sufficient quantity and quality of DNA.

There are three aims of this study, which was completed in collaboration with mother and baby pairs from women who took part in the OPPTIMUM trial ^11^. Firstly, to pilot the method of postal recruitment and sample collection, return and processing. Secondly, to confirm that maternally collected saliva samples, particularly from infants, provide sufficient high quality DNA yield. Thirdly, to assess the agreement of self-recalled pregnancy and birth details in a questionnaire, including essential information for preterm birth genetic association studies, compared with prospectively collected OPPTIMUM trial data.

## Methods

### Participants

**Figure 1.**
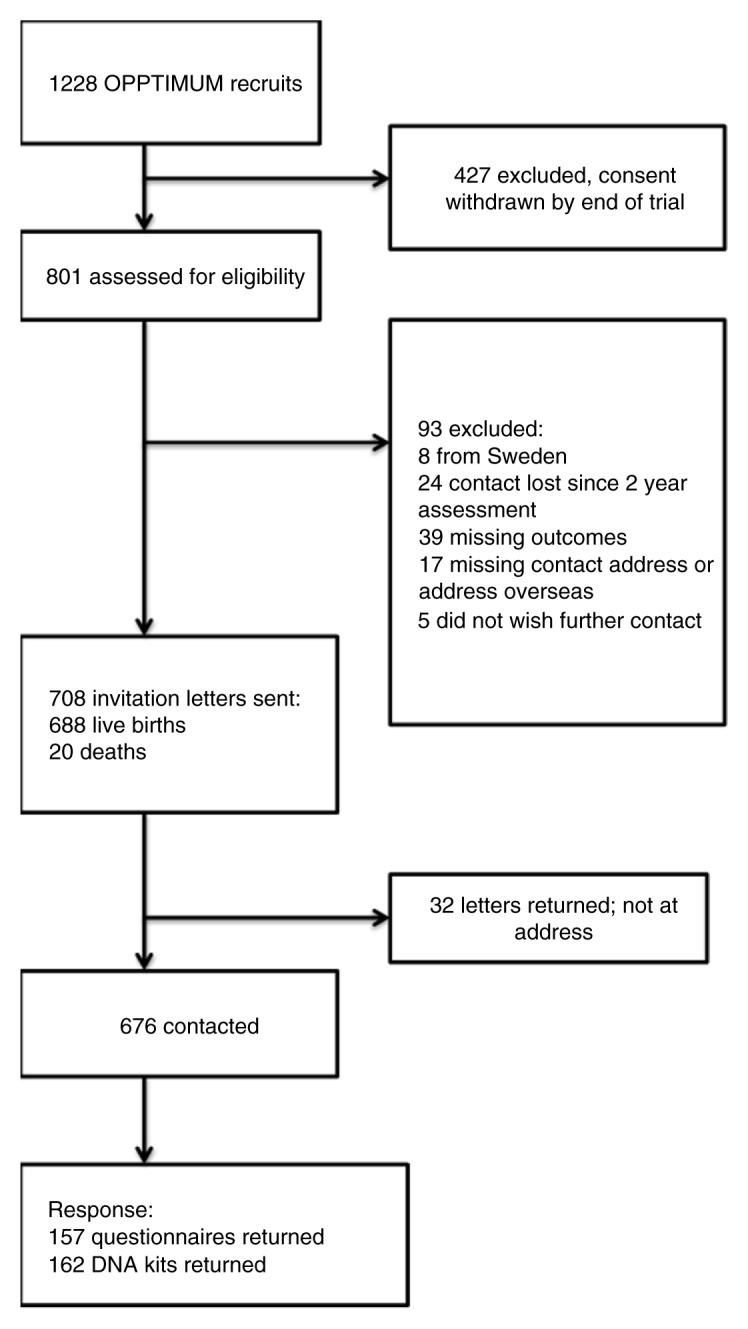
Study flow diagram of recruitment.

We included UK based participants from the OPPTIMUM trial, a double-blind, randomised, placebo-controlled trial investigating the effect of vaginal progesterone on pregnancy and infant outcomes in women at high risk of spontaneous PTB ( https://doi.org/10.1186/ISRCTN14568373) ^11^. OPPTIMUM recruited from 65 UK National Health Service (NHS) Hospitals and 1 Swedish hospital between February 2009 and April 2013. The inclusion criteria for the OPPTIMUM trial were: high risk for PTB, gestation established by scan at ≤ 16 weeks to ensure that estimated date of delivery is accurate, signed consent form and aged 16 years or older (no upper age limit). Exclusion criteria for the OPPTIMUM trial were: known significant congenital structural or chromosomal fetal anomaly, known sensitivity, contraindication or intolerance to progesterone, suspected or proven rupture of the fetal membranes at the time of recruitment, multiple pregnancy, prescription or ingestion of medications known to interact with progesterone and women who were prescribed progesterone who took progesterone beyond 18 weeks gestation (See extended data: Appendix 2 ^12^). For this study, we excluded women who: had withdrawn their consent from the OPPTIMUM trial, those of whom we had no contact details, those whose babies had died subsequent to the 2-year follow-up period, and those who were recruited in Sweden ( Figure 1).

### Recruitment

Potential participants were sent a letter of invitation to the “OPPTIMUM genetics” study from 20 ^th^ April 2015 to 23 ^rd^ July 2015 (see extended data ^12^). Women who had a stillbirth, neonatal or infant death were sent an alternate letter of invitation (see extended data ^12^). Women were asked to reply by email, phone, text or post to indicate their interest in participating. The study lasted for 12 months after the last recruitment pack was sent out.

### Sample collection

Women who responded positively to the invitation letter were sent a recruitment pack (see extended data: Appendix 3 ^12^). Women who had a live infant were invited to provide a saliva sample from themselves and their baby born whilst participating in the OPPTIMUM study; for those women who had experienced infant loss, an alternative pack was sent with a collection kit for themselves only. The recruitment packs contained a recruitment letter, patient information leaflet, maternal consent form (2 copies), child assent form (if child over 4 years old), instructions for saliva sample collection for mother and maternal saliva sample collection kit (Oragene OG-500, DNA Genotek), a clinical data questionnaire and a postal return kit (postage paid; see extended data ^12^). Where appropriate, instructions for saliva sample collection for baby, and an infant saliva sample collection kit (Oragene OG-575, DNA Genotek) was included. If the participant’s baby had died, they were invited to provide a saliva sample from themselves, and an alternative recruitment pack was sent (see extended data: Appendix 3 ^12^). Women who had not returned the recruitment pack after 6–8 weeks were sent a single reminder letter.

### Questionnaire design and application

The questionnaire was designed by the OPPTIMUM trial team to meet the minimum data required for a study of PTB ^13^ and was piloted in 20 postpartum women. It included questions on gestation at birth, maternal age at birth, birth method, labour onset, membrane rupture, maternal smoking, non-prescription drug use and alcohol intake, as well as the number of previous pregnancies and maternal ethnic origin (see extended data: Appendix 1 ^12^). Participants were asked to answer the questions in relation to their pregnancy during the OPPTIMUM trial.

### Receipt, processing and storage of samples

The Edinburgh Clinical Research Facility Genetics Core received and processed the samples. Samples were identified by the OPPTIMUM trial number, with a suffix for mother and baby and labelled with details readable by barcode scanner.

### DNA extraction and validation

DNA was extracted from Oragene OG-500 (adults) and OG-575 (babies) saliva kits using Oragene prepIT (PT-L2P-5) extraction kit (supplied by DNA Genotek). DNA yield was measured by Qubit (ThermoFisher). Samples were genotyped on a panel of Taqman single nucleotide polymorphisms (SNPs) using the QuantStudio12K Flex and analysed using QuantStudio v1.2.2 software. Samples from mothers were genotyped using autosomal SNPs rs6427699, rs4751955, rs11083515, rs7588807, rs10938367 & rs10869955. Samples from babies were run on the same six autosomal SNPs and an additional three SNPs from the Y-chromosome to determine sex ( rs2032598, rs768983 & rs3913290). An aliquot of DNA was normalised in plates for future analysis. DNA samples were transferred for storage and used as part of the Edinburgh Reproductive Tissues Biobank (REC reference 09/S0704/3).

### Data analysis

For this study, data was transcribed from the questionnaires and appropriate information was obtained from the trial database. Patient identifiable information was removed and trial data was correlated with the corresponding questionnaire through randomised OPPTIMUM trial numbers. In keeping with the Caldicott principles ^14^, access to trial data was only granted to members of the research team, and stored on a password protected database on a secure server (University of Edinburgh). The concordance between self-recalled birth details and prospective trial birth details was then analysed for gestation at birth, maternal age at birth, mode of birth, onset of labour, smoking, non-prescription drug use, alcohol use and number of previous pregnancies. The concordance for PPROM was analysed in women who had a PTB. Concordance was measured using Cohen’s kappa coefficient. The kappa ( *k*) statistic measures the extent of exact agreement, adjusting for chance agreement with values greater than 0.75 representing excellent concordance, values of 0.40 to 0.75 representing moderate concordance and values less than 0.40 representing poor concordance ^15^. In addition, Spearman’s rho ( *r*) correlation coefficient was calculated for each variable.

We pre-specified that a participation rate of ≥ 50% would be acceptable and *k* > 0.75 would be sufficient for phenotyping PTB. These values were chosen by the research team as previous studies of self-collected DNA samples have had participation rates above 50% ^16, 17^ and accurate recall using patient questionnaires would be necessary for any future large scale studies using this design.

### Statistical analysis

All data was analysed using IBM Statistical Package for the Social Sciences (SPSS) Version 22. Normally distributed data was analysed using a t-test. The Fishers exact test was used for proportional data. A p-value of <0.05 was considered significant.

### Ethical opinion

This study was awarded a favourable ethical opinion by the regional ethics committee (REC reference 14/SS/0086).

## Results

### Recruitment rate and participant demographics

**Figure 2.**
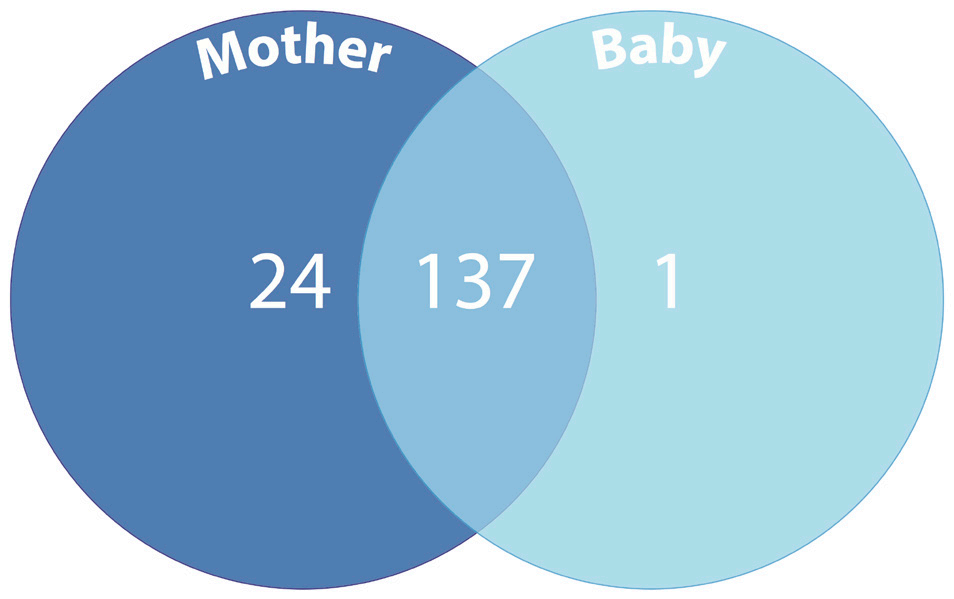
Venn diagram demonstrating DNA samples received: 24 mother only samples, 1 baby only sample and 137 mother and baby paired samples.

**Figure 3.**
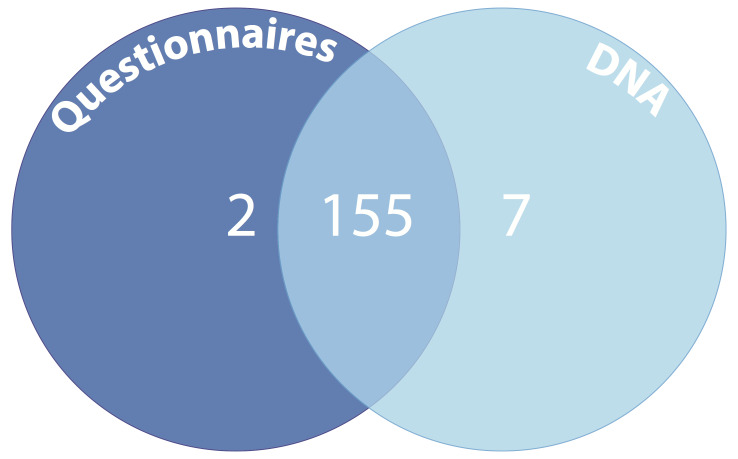
Venn diagram demonstrating the overlap of number of questionnaires received and number of DNA samples received: 2 respondents returned the questionnaire without DNA samples, 7 returned DNA samples without a questionnaire and 155 returned both DNA samples and questionnaire.

In total, 708 women were contacted. From these, 157 questionnaires were returned, a participation rate of 22%. Overall, 299 DNA sample kits were received (137 mother and baby paired samples, 24 mother only samples, 1 baby only sample) - a participation rate of 162/708 (23%) (see Figure 1 and Figure 2). Seven participants returned a DNA sample kit without a questionnaire and 2 participants returned the questionnaire without a DNA sample. One participant was recruited twice to the OPPTIMUM trial and so returned a questionnaire for each pregnancy, a mother and baby paired sample from her first child and a baby only sample from her second child (See Figure 3). See underlying data for all data collected ^12^.

The demographics of the questionnaire respondents are shown in Table 1. To determine if these 157 women were representative of the OPPTIMUM trial population the demographics of the participants in this pilot study were compared to that of the entire cohort of OPPTIMUM trial participants. This highlighted that the pilot study participants were significantly older, more educated, had a higher proportion of white participants, and a lower proportion of black participants compared with the entire OPPTIMUM cohort ( Table 1).

**Table 1.** Demographics of study participants.

	Pilot study		OPPTIMUM trial cohort	
	N	n (%) or mean (SD)	N	n (%) or mean (SD)
Age (years)	157	32.8 (5.5) *	1225	31.5 (5.7)
Smoking	157	14 (9%)	1220	63 (5%)
Alcohol	157	9 (6%)	1223	63 (5%)
Drug use	157	1 (0.6%)	1223	17 (1%)
Years in full-time education	147	14.4 (3.1) **	1122	13.5 (3.0)
Ethnic group				
White	157	139 (89%) **	1224	895 (73%)
Black	157	8 (5%) **	1224	180 (15%)
Asian	157	6 (4%)	1224	104 (8%)
Mixed	157	3 (2%)	1224	28 (2%)
Other	157	0 (0%)	1224	17 (1%)
Any previous pregnancy	154	147 (95%)	1224	1172 (96%)
History of preterm birth (any)	154	121 (79%)	1223	966 (79%)

Demographics of participants in the pilot study group (taken from OPPTIMUM trial data) versus the complete OPPTIMUM trial cohort. Each field was analysed for statistical differences between these two groups. SD = standard deviation. * = p<0.05, ** = p<0.001, *** = p<0.0001

### DNA extraction and quality assessment

DNA was extracted from 299 saliva samples. All 161 samples from the mother provided DNA with median yield 59.0µg (0.4-148.9µg). Two baby samples failed and provided no DNA. The remaining 136 baby samples had a median yield 11.5µg (0.1-102.7µg) ( Table 2). Samples were genotyped using a panel of six autosomal Taqman SNPs and were deemed suitable for genomic analysis if they successfully genotyped on five or more SNPs. 156 of the 161 samples (96.9%) from the mother successfully genotyped (one sample failed on four SNPs, three samples failed on five SNPs and one sample failed on all six SNPs). 131 of the 136 baby samples (96.3%) successfully genotyped (three samples failed on five SNPs and two samples failed on all six SNPs) ( Table 2). For those genotypes that were homozygous in the mother and the child we were able to demonstrate Mendelian consistency. Across the 6 autosomal SNPs there were 204 genotype calls we could compare. 201 showed Mendelian consistency. 3 genotype calls were inconsistent.

**Table 2.** Results of DNA analysis and genotyping from mother and baby self-collected saliva samples.

	Mother samples (n=161)	Baby samples (n=138)
Number of samples suitable for analysis	161 (100%)	136 (98.6%)
Median DNA yield in μg (range)	59.0 (0.4-148.9)	11.5 (0.1-102.7)
Number of samples successfully genotyped using 6 autosomes	156/161 (96.9%)	131/136 (96.3%)
Number of samples correctly identifying sex		121/124 (97.6%)

The baby samples were additionally genotyped on three Y-chromosome SNPs. Samples that successfully called on five or six autosomal SNPs were checked for sex, with samples having a no-call on all three Y-chromosomes SNPs determined as female and samples having a call on all three Y-chromosome SNPs determined as male. Samples having a positive call on only one or two Y-chromosome SNPs were assigned to ‘unknown’, of which 5 of 131 samples were assigned. Additionally, 2 samples did not have matched trial data to verify sex. In total, 121 of 124 samples (97.6%) correctly identified the sex of the baby; 3 samples which were assumed female from genotyping were male ( Table 2).

### Concordance and correlation of self-recalled birth details and prospective trial birth details

Concordance of self-recalled birth details and prospective trial birth details for each variable are displayed in Table 3. Concordance using Cohen’s kappa was excellent ( *k* > 0.75) in 4 out of 10 key fields for phenotyping PTB (mode of delivery, labour onset, ethnicity and maternal age at birth). Concordance was moderate (0.4 < *k* < 0.75) for smoking status, gestation at birth and number of previous pregnancies, and poor ( *k* < 0.4) for alcohol use and drug use. The concordance for PPROM was assessed in only 11 cases, where PPROM led to preterm birth. Only 6 participants out of the 11 cases recalled PPROM prior to having a preterm birth. It was not possible to calculate Cohen’s kappa for this group as the trial cohort had only one variable, but Spearman’s rho correlation coefficient was weak at 0.224.

**Table 3.** Table displaying Cohen’s kappa coefficient as a measure of concordance and Spearman’s rho coefficient as a measure of correlation of prospective trial birth details with self-recalled pregnancy and birth details. A p-value of <0.01 is statistically significant.

	N	Missing self-recall data	Missing prospective trial data	Cohen’s kappa (p value)	Spearman’s rho (p value)
**Mode of delivery**	157		1 (0.6%)	0.974 (0.000)	0.956 (0.000)
**Labour onset**	157	1 (0.6%)	1 (0.6%)	0.839 (0.000)	0.822 (0.000)
**Ethnicity**	157	1 (0.6%)		0.812 (0.000)	0.868 (0.000)
**Maternal age at birth**	157	1 (0.6%)		0.797 (<0.001)	0.980 (0.000)
**Smoking status**	157			0.749 (0.000)	0.751 (0.000)
**Gestation at birth**	157	1 (0.6%)	3 (2%)	0.742 (0.000)	0.908 (0.000)
**Number of previous pregnancies**	157			0.536 (0.000)	0.686 (0.000)
**Alcohol use**	157	1 (0.6%)		0.297 (0.000)	0.368 (0.000)
**Drug use**	157	1 (0.6%)	1 (0.6%)	0.012 (0.716)	-0.029 (0.718)
**Preterm premature rupture of** **membranes**	11			Not possible	0.224 (0.121)

Correlation measured using Spearman’s rho ( *r*) was very strong ( *r =* 0.8 - 1.0) in five fields for phenotyping PTB (mode of delivery, labour onset, ethnicity, maternal age at birth and gestation at delivery). Spearman’s rho was strong ( *r* = 0.60 – 0.79) for smoking status and number of previous pregnancies, weak (r = 0.20 – 0.39) for alcohol use and very weak ( *r* = 0.00-0.19) for drug use.

### Smoking status, non-prescription drug use, alcohol consumption

There was no statistical difference found between the number of smokers reported in prospective trial data and self-recalled data (p=0.838). The difference in non-prescription drug use between prospective trial data and self-recalled data was statistically significant (p=0.0001); 18/157 (11%) of women recorded as no drug use in prospective trial data classified themselves as having used non-prescription drugs during their pregnancy. A subset of these women (10/18, 56%) included the names of the drugs used; these included paracetamol, aspirin, folic acid, codeine and “indigestion medication”. The difference between alcohol consumption in prospective trial data and self-recalled data was statistically significant (p=0.0005); 23/148 (16%) women recorded as non-drinkers in prospective trial data self-recalled use of alcohol during their pregnancy.

## Discussion

The primary aim of this study was to pilot postal donation of self-collected DNA samples from postpartum women and their babies. The recruitment rate of 23% was below our pre-specified response rate of ≥ 50% and compares poorly with other studies investigating self-collection of DNA which have had participation rates greater than 50% ^16, 17^. Only one reminder was sent - it is possible that further reminders might have increased the participation rate. We recruited participants from a cohort of women who had previously taken part in the OPPTIMUM trial. Although this limited our study population, the main advantages were that consent had already been obtained and that high quality prospectively collected data on demographics and birth outcomes was available for validation.

The Preterm Birth Genome Project investigated multiple methods of DNA collection from mother and baby pairs (whole blood, blood spot, buccal, saliva) from four countries and found that samples were not affected by transportation methods and that salivary samples provided an adequate yield of DNA, superior to buccal swabs ^18^. Similar studies of self-collected saliva samples report DNA yields of > 70% ^19, 20^. Our study shows that postal self-collected saliva samples from mothers and babies were of sufficient quality for genetic analysis but yields were variable.

We also aimed to determine the concordance between self-recalled birth details and prospective trial birth details with a view to determine if postal questionnaires are a valid method of phenotyping PTB. We demonstrate that concordance was above the pre-specified *k* > 0.75 in only four out of ten questions. It is very unlikely that the prospectively collected OPPTIMUM trial data was erroneous. The study was carried out to rigorous clinical trial standards with a pre-defined data dictionary and training of all research staff contributing to data collection. There was regular trial data monitoring from the sponsor ensuring data quality and consistency with checking of source data.

Labour onset and membrane rupture are important features which differentiate spontaneous PTB from PTB following PPROM and medically indicated PTB. This is crucial, as the underlying genetic basis for each phenotype is potentially different. In this dataset, concordance for labour onset was excellent ( *k* = 0.839). The correlation between self-recalled details and trial data for the occurrence of PPROM in women who had a PTB was weak suggesting that PPROM is not accurately recalled. However, we recognise that this is a very small subset of women and a larger number would be required to accurately assess this finding.

In contrast to the moderate level of concordance for smoking status, the concordance between prospective trial data and self-recalled data was poor with regards to non-prescription drug use and alcohol intake: a much higher level of alcohol consumption was reported in self-recalled data than in prospective trial data. Interestingly, the discrepancies between the reporting of smoking, alcohol and non-prescription drug use are in both directions. This highlights differences in public perception and willingness to disclose such information whilst in a research trial or in an anonymised questionnaire. The inconsistencies in reporting of non-prescription drug use were mainly due to several women self-recalling use of ‘over the counter’ medications such as paracetamol. This is in contrast to the OPPTIMUM trial definition of non-prescription drug use which included: heroin, cocaine or abuse of prescribed drugs such as benzodiazepines. This finding is most likely due to a misinterpretation of the question and highlights the importance of accurate wording in similar questionnaire studies.

In conclusion, this feasibility study shows that women can successfully collect DNA from themselves and their babies, but overall yields were variable. Yield from babies was lower than mothers due to the lower amount of saliva collected with the Oragene OG-575. Taqman genotyping showed the samples were suitable for variant calling and checking sex. The low yields, particularly for the baby samples, would make some methods of genomic analysis challenging, such as exome sequencing, but with the development of low-input kits even these methods may be suitable. Consideration should be given to the genetic analysis to be performed when deciding on a collection method.

We found postal post-partum participation rates are low and there were significant discrepancies between self-recall of pregnancy and birth details and prospective birth details as recorded in the OPPTIMUM trial dataset. We conclude that information gathered from postal questionnaires is insufficient to accurately phenotype PTB and clinical data collection from medical records needs to be an integral part of any future study design into the genetics of preterm birth.

## Data availability

### Underlying data

Figshare: Postal recruitment for genetic studies of preterm birth: A feasibility study. https://doi.org/10.6084/m9.figshare.7887083 ^12^

This project contains the following underlying data:

- Figshare1. DNA data.csv (Dataset showing total DNA yield for all self-collected samples)
- Figshare2. OPPTIMUM trial data.csv (Prospective trial data)
- Figshare3. Questionnaire data.csv (Data from participant questionnaires)
- Figshare4. Comparison results of y markers_281019_BABY.csv (Results of determining sex of baby from saliva samples)
- Figshare5. Taqman Genotyping Report_291019.pdf (Report detailing results of Taqman genotyping)
- Genotyping results for 6 x SNPs using mother and baby samples (individual files for Taqman genotyping results for mother and baby for each SNP: rs6427699, rs4751955, rs11083515, rs7588807, rs10938367 & rs10869955)

### Extended data

Figshare: Postal recruitment for genetic studies of preterm birth: A feasibility study. https://doi.org/10.6084/m9.figshare.7887083 ^12^

This project contains the following extended data:

- Appendix 1. Participant questionnaire (example of participant questionnaire)
- Appendix 2. OPPTIMUM trial inclusion and exclusion criteria (list of inclusion and exclusion criteria for recruitment to the OPPTIMUM trial)
- Appendix 3. List of recruitment pack contents (list of recruitment pack contents for women who had a live birth, and for women who had a stillbirth, neonatal or infant death)
- OPPTIMUM Genetics invitation letters (invitation letters to participate in OPPTIMUM Genetics study, one for women who had a live birth, one for women who had a stillbirth, neonatal or infant death)
- Recruitment pack letters (letters included in recruitment pack, one for women who had a live birth, one for women who had a stillbirth, neonatal or infant death)
- OPPTIMUM genetics patient information leaflets (patient information leaflets, one for women who had a live birth, one for women who had a stillbirth, neonatal or infant death)
- OPPTIMUM genetics consent forms (participant consent form, one for women who had a live birth including consent for her baby or child to participate, one for women who had a stillbirth, neonatal or infant death)
- OPPTIMUM genetics child assent form (for children over 5 years of age to assent to participation in study)
- Saliva sample collection instructions (instructions on how to collect saliva sample from participant and from baby or child using Oragene saliva kits)

Data are available under the terms of the Creative Commons Zero “No rights reserved” data waiver (CC0 1.0 Public domain dedication).
