# Supplementary material for: Postal recruitment for genetic studies of preterm birth: A feasibility study
Source: Wellcome Open Res. 2020 Jun 30;5:26. Originally published 2020 Feb 12. [Version 2] doi: 10.12688/wellcomeopenres.15207.2 (PMC7160603; doi:10.12688/wellcomeopenres.15207.2)
Supplement: Peer review history

# Peer review: Reviewer response for version 1

**DOI:** 10.21956/wellcomeopenres.16595.r38279

**Published:** 2020-04-27

**Authors:** Ge Zhang

**Affiliations:** 1 Division of Human Genetics, The Center for Prevention of Preterm Birth, Perinatal Institute, Cincinnati Children's Hospital Medical Center, Cincinnati, OH, USA

This pilot study evaluated the feasibility of retrospective collection of DNA samples and self-recalled phenotype data for genetic studies of preterm birth. The study demonstrated the DNA samples were sufficient for genetic analysis but the self-recalled phenotype data was inaccurate. The reviewer has major concern with using concordance in evaluating the consistency of phenotype data.

1. Introduction: ref 11 demonstrated that successful genetic studies of gestational duration and preterm birth require large sample sizes. It did not study the genetic or pathophysiological differences among sPTB, PPROM and indicated PTB.
2. Methods (Data analysis): The calculation of concordance should be explicitly explained.
3. Methods (Data analysis): In addition to concordance, the authors should check correlations (especially for quantitative variables and binary variables) between the self-recalled data and data collected from the clinical trial.
4. Results (first paragraph): The authors may provide Venn diagrams to show the overlapping of DNA samples between mothers and children [24 (137) 1] and overlapping between questionnaires and DNA samples [2 (155 ) 7].
5. Results (second paragraph and Table 1): It should be clarified that the numbers [n(%)] were calculated based on trial data even for the pilot study.
6. Results (DNA quality assessment): The author should present Mendelian consistency between mothers and their child.
7. Results (Table 3): concordance of PPROM was calculated as 6/11 (55%), which might be incorrect (please also see my comment 2).
8. Results (Please also see my comments 2 and 3): It seems to the reviewer that concordance is not an adequate measure to evaluate the consistency between self-recalled data and the data collected during the trial. For example, only 1 mother in the pilot study was recorded having non-prescription drugs; but 18 recorded as no drug use recalled themselves as having used non-prescription drugs. This information is totally inconsistent and misleading but still the concordance is 88% = (155-(18+1))/155. The same is true for alcohol consumption. As suggested by my comment 3, the authors should check correlations or present Venn diagrams for every categorical variable to show the complete picture of relationships between the two data sets.

Is the work clearly and accurately presented and does it cite the current literature?

Partly

If applicable, is the statistical analysis and its interpretation appropriate?

Partly

Are all the source data underlying the results available to ensure full reproducibility?

Yes

Is the study design appropriate and is the work technically sound?

Partly

Are the conclusions drawn adequately supported by the results?

Partly

Are sufficient details of methods and analysis provided to allow replication by others?

Partly

Reviewer Expertise:

Statistical genetics

I confirm that I have read this submission and believe that I have an appropriate level of expertise to state that I do not consider it to be of an acceptable scientific standard, for reasons outlined above.

# Response

**Published:** 2020-06-23

**Authors:** Oonagh Keag

**Affiliations:** NHS Lothian, UK

**1. Introduction: ref 11 demonstrated that successful genetic studies of gestational duration and preterm birth require large sample sizes. It did not study the genetic or pathophysiological differences among sPTB, PPROM and indicated PTB.**

Thank you for your comment. Where ref 11 was cited, it should have been ref 10 – the Pennell group highlights the important differences between spontaneous PTB, spontaneous PTB with PPROM and medically indicated PTB. This group stresses the necessity for accurate phenotyping. This has been corrected in the revised version.

**2. Methods (Data analysis): The calculation of concordance should be explicitly explained.**

Concordance was analysed by directly comparing the trial data to the participant questionnaire responses and determining the percentage of responses which were the same. SPSS was used to calculate the 95% confidence intervals. The authors are aware of the use of Cohen’s kappa coefficient in measuring concordance and have calculated this for each variable. These results are similar to the direct observation of concordance except for in drug use, alcohol use and for PPROM. The kappa statistic measures the extent of exact agreement, adjusting for chance agreement with values greater than 0.75 representing excellent concordance; values of 0.40 to 0.75 representing moderate concordance; and values less than 0.40 representing poor concordance. (Ref: Landis JR, Koch GG: The measurement of observer agreement for categorical data. Biometrics. 1977, 33 (1): 159-174. 10.2307/2529310.) The authors agree that the kappa statistic is more reflective of the agreement of the trial and questionnaire data and so the direct comparison method has been removed in version 2 and the table updated (see Table 3).

**3. Methods (Data analysis): In addition to concordance, the authors should check correlations (especially for quantitative variables and binary variables) between the self-recalled data and data collected from the clinical trial.**

Spearman correlation coefficients have been calculated for each variable and are displayed in Table 3 of the revised version.

**4. Results (first paragraph): The authors may provide Venn diagrams to show the overlapping of DNA samples between mothers and children [24 (137) 1] and overlapping between questionnaires and DNA samples [2 (155 ) 7].**

Thank you for your suggestion of Venn diagrams to describe our results. We have added these to the revised version as Figures 2 and 3.

**5. Results (second paragraph and Table 1): It should be clarified that the numbers [n(%)] were calculated based on trial data even for the pilot study.**

We can clarify that the demographics of the questionnaire respondents described in Table 1 are based on the trial data.

**6. The author should present Mendelian consistency between mothers and their child.**

We can only do this by looking at those genotypes that are homozygous in the mother and the child, as we have no paternal information. On this basis, across the 6 autosomal SNPs there were 204 genotype calls we could compare. 201 showed Mendelian consistency. 3 genotype calls were inconsistent (presumably these were then 3 mic-calls). Added to the sex-chromosome data where all samples were correctly allocated gender we think we have shown accuracy of sample collection and genotyping.

**7. Results (Table 3): concordance of PPROM was calculated as 6/11 (55%), which might be incorrect (please also see my comment 2).**

The concordance for PPROM was assessed in only 11 cases, where PPROM led to preterm birth. 6 participants out of the 11 cases recalled PPROM prior to having a preterm birth, 6/11 = 55%. It was not possible to calculate Cohen’s kappa for this group as the trial cohort had only one variable.

**8. (Please also see my comments 2 and 3): It seems to the reviewer that concordance is not an adequate measure to evaluate the consistency between self-recalled data and the data collected during the trial. For example, only 1 mother in the pilot study was recorded having non-prescription drugs; but 18 recorded as no drug use recalled themselves as having used non-prescription drugs. This information is totally inconsistent and misleading but still the concordance is 88% = (155-(18+1))/155. The same is true for alcohol consumption. As suggested by my comment 3, the authors should check correlations or present Venn diagrams for every categorical variable to show the complete picture of relationships between the two data sets.**

Thank you for your comments. The authors agree that in the alcohol use and non-prescription drug use groups there are significant discrepancies which aren’t so apparent when concordance is presented. This does become apparent when Cohen’s kappa is calculated; its value is low in both groups, as seen in Table 3 of the revised version. This is the same when correlation is measured in these groups using Spearman’s coefficient. The authors attempt to address these inconsistencies in the discussion, highlighting the potential misunderstanding or misinterpretation of the drug use question, evident from the free text answers including over the counter medications such as paracetamol.

# Peer review: Reviewer response for version 1

**DOI:** 10.21956/wellcomeopenres.16595.r38338

**Published:** 2020-04-15

**Authors:** Kelly-Ann Eastwood

**Affiliations:** 1 Centre for Public Health, Institute of Clinical Sciences, Block B, Royal Victoria Hospital, Queen’s University Belfast, Belfast, UK

This is an interesting study investigating feasibility of self-collected postnatal maternal and neonatal saliva for DNA extraction. The paper is well written and of interest to clinicians and researchers.

 The authors have clearly discussed limitations of the study, including recruitment rate (23%) and poor concordance between self-recalled birth details and prospective trial data.

 I wonder if recruitment rate may have in fact been bolstered by previous participation in the OPTIMMUM study. This could have implications in a real world clinical setting. Regarding collection of saliva samples; median DNA yield demonstrated a large range for both mother and neonatal samples. Going forward, how could integrity of sampling technique be assured?

Is the work clearly and accurately presented and does it cite the current literature?

Yes

If applicable, is the statistical analysis and its interpretation appropriate?

I cannot comment. A qualified statistician is required.

Are all the source data underlying the results available to ensure full reproducibility?

Yes

Is the study design appropriate and is the work technically sound?

Yes

Are the conclusions drawn adequately supported by the results?

Yes

Are sufficient details of methods and analysis provided to allow replication by others?

Yes

Reviewer Expertise:

Academic Clinical Lecturer in O&G, sub-specialty trainee in maternal and fetal medicine. Research interests include pre-eclampsia, pre-conception care and maternal obesity.

I confirm that I have read this submission and believe that I have an appropriate level of expertise to confirm that it is of an acceptable scientific standard.

# Peer review: Reviewer response for version 1

**DOI:** 10.21956/wellcomeopenres.16595.r37902

**Published:** 2020-03-30

**Authors:** Marie Anne Ledingham

**Affiliations:** 1 Ian Donald Fetal Medicine Unit, Queen Elizabeth Hospital, Glasgow, UK

This study examines the feasibility of postnatal recruitment for analysis of genetic factors associated with preterm birth. It is methodologically appropriately designed and the conclusions drawn by the authors seem appropriate. This study highlights the difficulty involved in reliance upon recruitment into studies remotely and the problems with self reporting of outcomes which often don't tally with correct medical diagnosis.

Is the work clearly and accurately presented and does it cite the current literature?

Yes

If applicable, is the statistical analysis and its interpretation appropriate?

I cannot comment. A qualified statistician is required.

Are all the source data underlying the results available to ensure full reproducibility?

Yes

Is the study design appropriate and is the work technically sound?

Yes

Are the conclusions drawn adequately supported by the results?

Yes

Are sufficient details of methods and analysis provided to allow replication by others?

Yes

Reviewer Expertise:

Maternal and Fetal medicine, preterm labour, multiple pregnancy

I confirm that I have read this submission and believe that I have an appropriate level of expertise to confirm that it is of an acceptable scientific standard.

# Peer review: Reviewer response for version 1

**DOI:** 10.21956/wellcomeopenres.16595.r37901

**Published:** 2020-02-19

**Authors:** Tessa Homfray

**Affiliations:** 1 South West Thames Regional Genetics ServiceSouth West Thames Regional Genetics Service, St George's University Hospitals NHS Foundation Trust, London, UK; 2 Harris Birthright Unit, King's College Hospital, London, UK

- I am uncertain exactly when these women were recruited. The OPPTIMUM study was 2013 and therefore this was sometime later that this was attempted which would I suspect lead to the poorer recall than if this had been attempted later.
- The amount of baby saliva DNA would be too small to be of much use I suspect. 
- I didn't see any evidence confirming maternity of the fetal samples which would indicate if they had been collected correctly.
- This is a proof of principle study which suggests this is not a good method of obtaining genetic material. On this basis I think the article should be much shorter.

Is the work clearly and accurately presented and does it cite the current literature?

Yes

If applicable, is the statistical analysis and its interpretation appropriate?

Partly

Are all the source data underlying the results available to ensure full reproducibility?

Yes

Is the study design appropriate and is the work technically sound?

Partly

Are the conclusions drawn adequately supported by the results?

Partly

Are sufficient details of methods and analysis provided to allow replication by others?

Yes

Reviewer Expertise:

Consultant Medical Genetics with Particular interest in fetal medicine.

I confirm that I have read this submission and believe that I have an appropriate level of expertise to state that I do not consider it to be of an acceptable scientific standard, for reasons outlined above.

# Response

**Published:** 2020-06-23

**Authors:** Oonagh Keag

**Affiliations:** NHS Lothian, UK

**1. I am uncertain exactly when these women were recruited. The OPPTIMUM study was 2013 and therefore this was sometime later that this was attempted which would I suspect lead to the poorer recall than if this had been attempted later.**

Thank you for your comment. Although the OPPTIMUM trial finished recruitment in 2013, the study did not complete until July 2015 when we conducted the final childhood assessment at age 2. The trial team were in regular contact with many of the women for more than 5 years, on childhood birthdays and sending Christmas cards, newsletter and there was a facebook page with which many women engaged. As described in the methods, participants for this feasibility study were recruited in 2015, which would explain poorer recall in some areas. The reasons for using the OPPTIMUM participants was that consent had already been obtained for future contact and we had high quality prospectively collected data with which to compare questionnaire responses.

**2. The amount of baby saliva DNA would be too small to be of much use I suspect.**

Yes we have extracted low amounts but these have been used to confirm gender and parentage so not useless. It simply limits the amount we can do with the samples genetically. 

**3. I didn't see any evidence confirming maternity of the fetal samples which would indicate if they had been collected correctly.**

Thank you for your comment. We can only do this by looking at those genotypes that are homozygous in the mother and the child, as we have no paternal information. On this basis, across the 6 autosomal SNPs there were 204 genotype calls we could compare. 201 showed Mendelian consistency. 3 genotype calls were inconsistent (presumably these were then 3 mic-calls). Added to the sex-chromosome data where all samples were correctly allocated gender we think we have shown accuracy of sample collection and genotyping.

**4. This is a proof of principle study which suggests this is not a good method of obtaining genetic material. On this basis I think the article should be much shorter.**

Thank you for your comment. We have shown that postal recruitment was low and DNA yields were variable (particularly from babies) so future study designs need to take into consideration the yield required when planning how to analyse the DNA provided. One of our study’s aims was to assess the concordance between self-recalled pregnancy details and prospective trial data and the authors felt it necessary and interesting to discuss the discrepancies which were uncovered, highlighting the difficulties encountered when using postal questionnaires, and suggesting that this is not a reliable method to phenotype preterm birth.
